# Derivation and Characterization of Hepatic Progenitor Cells from Human Embryonic Stem Cells

**DOI:** 10.1371/journal.pone.0006468

**Published:** 2009-07-31

**Authors:** Dongxin Zhao, Song Chen, Jun Cai, Yushan Guo, Zhihua Song, Jie Che, Chun Liu, Chen Wu, Mingxiao Ding, Hongkui Deng

**Affiliations:** 1 Key Laboratory of Cell Proliferation and Differentiation of the Ministry of Education, College of Life Sciences, Peking University, Beijing, China; 2 Laboratory of Chemical Genomics, Shenzhen Graduate School of Peking University, The University Town, Shenzhen, China; KU Leuven, Belgium

## Abstract

The derivation of hepatic progenitor cells from human embryonic stem (hES) cells is of value both in the study of early human liver organogenesis and in the creation of an unlimited source of donor cells for hepatocyte transplantation therapy. Here, we report for the first time the generation of hepatic progenitor cells derived from hES cells. Hepatic endoderm cells were generated by activating FGF and BMP pathways and were then purified by fluorescence activated cell sorting using a newly identified surface marker, N-cadherin. After co-culture with STO feeder cells, these purified hepatic endoderm cells yielded hepatic progenitor colonies, which possessed the proliferation potential to be cultured for an extended period of more than 100 days. With extensive expansion, they co-expressed the hepatic marker AFP and the biliary lineage marker KRT7 and maintained bipotential differentiation capacity. They were able to differentiate into hepatocyte-like cells, which expressed ALB and AAT, and into cholangiocyte-like cells, which formed duct-like cyst structures, expressed KRT19 and KRT7, and acquired epithelial polarity. In conclusion, this is the first report of the generation of proliferative and bipotential hepatic progenitor cells from hES cells. These hES cell**–**derived hepatic progenitor cells could be effectively used as an *in vitro* model for studying the mechanisms of hepatic stem/progenitor cell origin, self-renewal and differentiation.

## Introduction

Human embryonic stem (hES) cells have the ability to grow infinitely while still maintaining the pluripotency required for differentiation into almost any cell type [1]. Thus, hES cells constitute a potential cell source for a variety of applications, such as studies of the fundamental mechanisms of lineage commitment and cell-based therapy in a broad spectrum of diseases. Among the different lineages that can be generated from hES cells, hepatic cells are of particular interest because the liver plays a major role in metabolism and has multiple functions, including glycogen storage, decomposition of red blood cells, plasma protein synthesis, and detoxification. A number of studies have demonstrated the feasibility of differentiating human or mouse ES cells into the hepatic lineage [2]–[6]. We have established a protocol for efficient production of hepatocytes by mimicking natural embryonic liver development *in vivo* [7]. During the differentiation process, we and other groups have observed that hepatocytes and cholangiocytes are generated concomitantly [3], [7], which suggests a common ancestor; that is, hepatic progenitor cells may exist. The existence of comparable hepatic progenitor cells in the ES differentiation process, however, has not been demonstrated. The properties and proliferation potential of these cells have not yet been characterized, and the mechanism of primary lineage transition has not been elucidated.

Hepatic progenitor cells serve as the major component of the hepatic parenchyma in early stages of liver organogenesis [8]. Studies of mouse and human embryonic development indicate that they are common progenitors of mature hepatocytes and biliary epithelial cells, the lineage commitments of which are determined around the mid-gestation stage [9]. Much research has been carried out on the development of *in vitro* culture systems for hepatic progenitor cells isolated from both human and mouse fetal livers [10]–[15]. Human hepatic progenitor cells exhibited phenotypic stability after extensive expansion [13] and, when placed in appropriate conditions, could differentiate into hepatocytes, which expressed ALB and stored glycogen, and into bile duct cells, which expressed KRT19 [12], [13]. Although the proliferation and bipotential capacity of hepatic progenitor cells have been demonstrated, the origin and function of hepatic progenitor cell populations are areas of ongoing debate [9]. The difficulty may be partly due to the shortage of material from early human embryos and undefined stages of development, given that hepatic progenitor cells have been directly separated only from human liver organs to date. Therefore, *in vitro* generation of hepatic progenitor cells based on a hES cell differentiation system offers a novel platform for further research on hepatic progenitor cells.

In this study, we first identified N-cadherin as a surface marker of hepatic endoderm cells for purification from hES cell**–**derivates, and generated hepatic progenitor cells from purified hepatic endoderm cells by co-culture with murine embryonic stromal feeders (STO) cells. These hepatic progenitor cells could expand and be passaged for more than 100 days. Interestingly, they co-expressed the early hepatic marker AFP and biliary lineage marker KRT7, suggesting that they are a common ancestor of both hepatocytes and cholangiocytes. Moreover, these progenitor cells could be expanded extensively while still maintaining the bipotential of differentiation into hepatocyte-like cells and cholangiocyte-like cells, as verified by both gene expression and functional assays. Therefore, this work offers a new *in vitro* model for studying liver development, as well as a new source for cell therapy based on hepatic progenitors.

## Results

### Identification of N-cadherin as a novel surface marker of hES cell–derived hepatic endoderm cells

**Figure 1:**
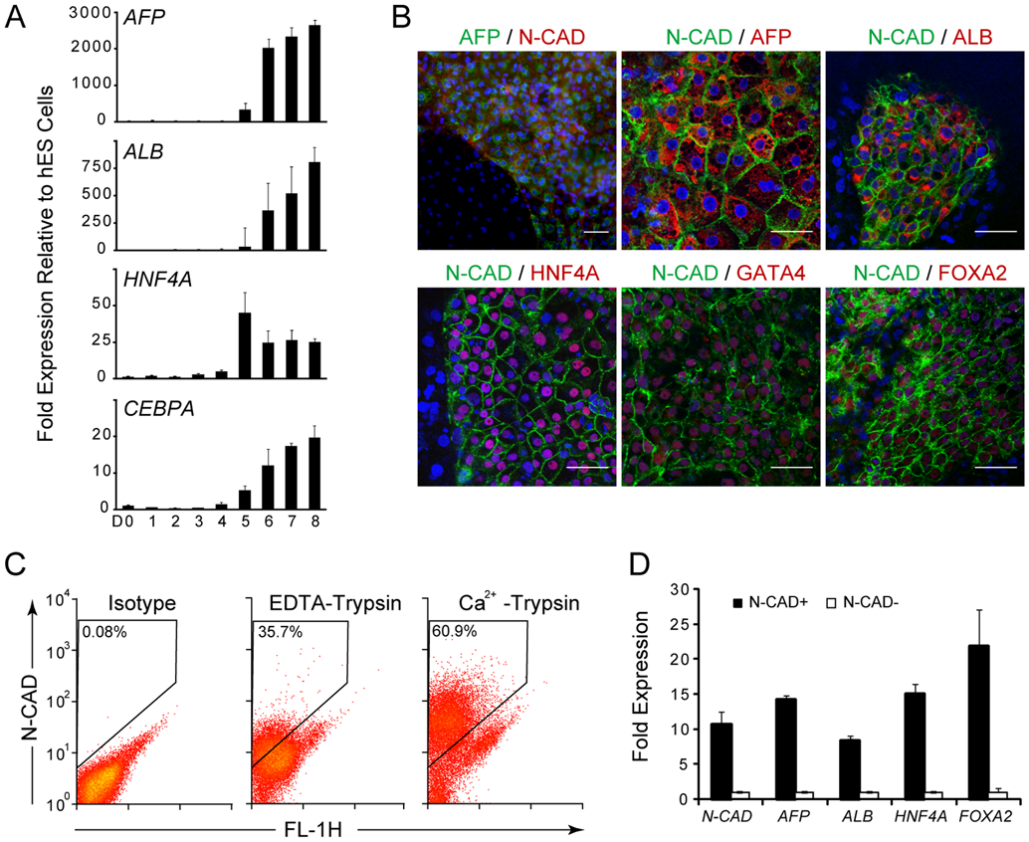
N-cadherin expression marks hepatic endoderm cells. (A) Temporal gene expression analysis of hepatic endoderm cells differentiated from human ES cells (quantitative RT-PCR). Expression level of the differentiated cells was calculated relative to undifferentiated hES cells. (B) Immunofluorescence showing that N-cadherin is co-expressed with the hepatic endoderm markers AFP, ALB, HNF4A, GATA4, and FOXA2 at day 8. The upper left image was taken using a fluorescence microscope, while the others were taken with a laser scanning confocal microscope. Scale bar = 50 µm. Cell nuclei are stained with DAPI (blue). (C) Flow cytometric isolation of N-cadherin-expressing hES cell–derived hepatic endoderm cells digested with trypsin and EDTA or with trypsin and Ca^2+^ at day 8. (D) Quantitative RT-PCR results showing elevated expression of hepatic marker genes in post-sorted N-cadherin^+^ cells. Y-axis, relative expression to *GAPDH*, then normalized to the N-cadherin^−^ cells.

We previously established a stepwise protocol to differentiate hES cells into hepatocytes by mimicking embryonic development [7]. We produced hepatic endoderm cells by using this protocol. hES cells were first exposed to Activin A for three days to induce definitive endoderm formation, and then were treated with BMP2 and FGF4 for another five days to induce hepatic endoderm cells. During this process, reverse-transcription (RT)-PCR was performed to assess the temporal gene expression of the hepatic marker genes *AFP*, *ALB*, *HNF4A* and *CEBPA*. All of these genes demonstrated similar expression patterns starting at around day 5 from the beginning of induction and reaching a maximal level at day 8 (Figure 1A), indicating the generation of hepatic endoderm.

To better analyze the properties of the hepatic endoderm cell population and eliminate possible interference from other cell lineages, we searched for a surface marker to purify hepatic endoderm cells from hES cell derivatives. We systematically tested a panel of putative hepatic progenitor cell surface markers, including CD29, CD34, CD49f, CD133, c-kit, c-met, Thy-1, N-cadherin, E-cadherin, EpCAM and NCAM (Table S1). Immunofluorescence using antibodies specific for N-cadherin revealed that nearly all cell labeling was restricted to AFP-expressing cells in the mixture of hES cell derivatives; moreover, no AFP-expressing cells lacked N-cadherin labeling. This result was confirmed by repeat tests using both fluorescence microscopy and laser scanning confocal microscopy (Figure 1B). Intracellular flow cytometry staining produced similar results, with co-expression of N-cadherin and AFP in a single cell (Figure S1). Further immunofluorescence analysis with confocal microscopy revealed concomitant expression of N-cadherin and the hepatic endoderm markers ALB, HNF4A, FOXA2 and GATA4 (Figure 1B).

**Figure 2:**
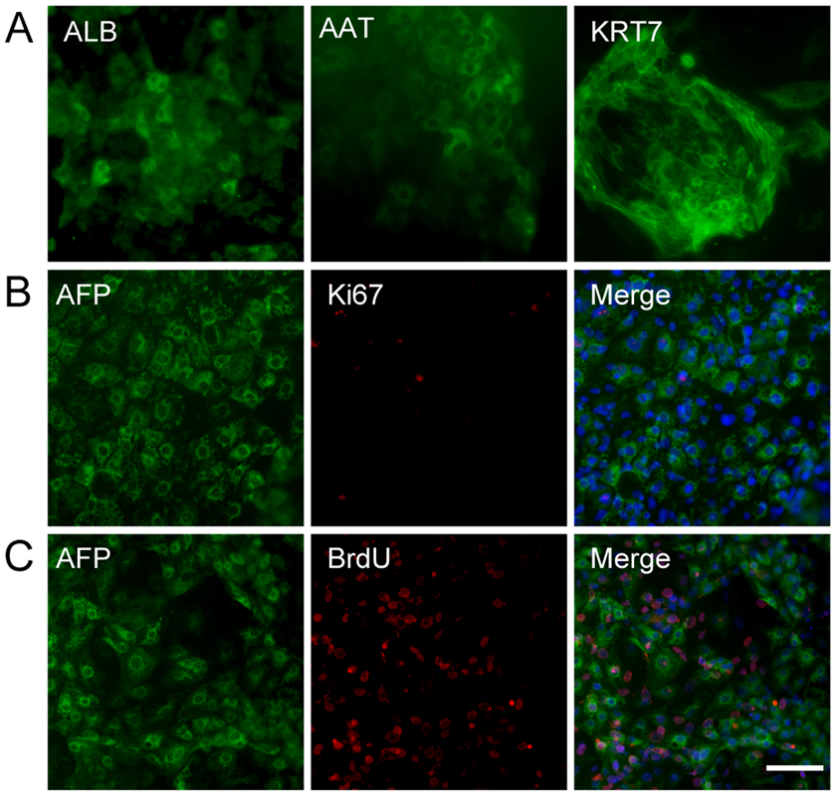
Characterization of sorted N-cadherin^+^ cells. (A) N-cadherin^+^ hepatic endoderm cells can further differentiate into hepatocyte-like cells and cholangiocyte-like cells. Immunostaining for ALB *(left)*, AAT *(middle)*, and KRT7 *(right)* in day 18 cultures generated from day 8 sorted cells. (B–C) Day 8 N-cadherin^+^ hepatic endoderm cells showed little proliferation potential, as demonstrated by Ki67 expression *(B)* and BrdU incorporation *(C)*. Note that most AFP^+^ cells were negative for BrdU. Scale bar = 50 µm.

To purify hepatic endoderm cells from hES cell derivatives, we set out to isolate the N-cadherin^+^ cell population and collected N-cadherin**^−^** cells for comparison. N-cadherin is a calcium-dependent cell-cell adhesion glycoprotein that is highly sensitive to trypsin treatment but can be efficiently protected from protease digestion by Ca^2+^ [16]. When hepatic endoderm cells were digested with 0.25% trypsin and 0.53 mM EDTA, most of the N-cadherin in the extracellular domain was cleaved and the cells were no longer recognized by the monoclonal antibody GC4 (Figure 1C, *middle*) [17]. In contrast, when hepatic endoderm cells were treated with 0.25% trypsin and 2 mM Ca^2+^ instead of EDTA to keep N-cadherin intact, a substantial portion of the population displayed positive staining for N-cadherin (60.9%±9.1%) by day 8 of differentiation (Figure 1C, *right*). Immunofluorescence of post-sorted cells revealed that the N-cadherin^+^ fraction consisted of >90% of the AFP-expressing cells, whereas few N-cadherin^−^ cells were AFP-positive (Figure S2). Further analysis by quantitative RT-PCR showed that the isolated N-cadherin^+^ cells were enriched for expression of the hepatic-specific genes *AFP*, *ALB*, *HNF4A* and *FOXA2* (Figure 1D). Additionally, this N-cadherin^+^ cell population could further mature into ALB- and AAT-expressing hepatocyte-like cells and KRT7-expressing cholangiocyte-like cells (Figure 2A) using a previously established protocol [7], while the N-cadherin^−^ cell population could not. Therefore, N-cadherin could serve as a surface marker of hepatic endoderm cells for purification from mixed hES cell derivatives.

### Generation and expansion of hepatic progenitor cells from N-cadherin^+^ hES cell–derived hepatic endoderm cells

In embryonic liver development, once hepatic specification is initiated and the liver bud is generated, hepatic progenitor cells greatly expand to generate the final volume of the liver [8]. When hES cell**–**derived hepatic endoderm cells were examined closely, they exhibited little proliferation potential. Staining for Ki67 and AFP at the end of the second stage revealed few AFP^+^ hepatic endoderm cells co-expressing Ki67 (Figure 2B). When BrdU was added to the differentiated cells over the entire 5 days of the hepatic endoderm generation stage, less than 5% of the AFP^+^ hepatic endoderm cells demonstrated BrdU incorporation (Figure 2C). Taken together, hES cell**–**derived hepatic endoderm cells differentiated rapidly without extensive expansion. This observation suggested that the culture conditions used may not have favoured the proliferation of hepatic progenitor cells and prompted us to search for better culture condition.

**Figure 3:**
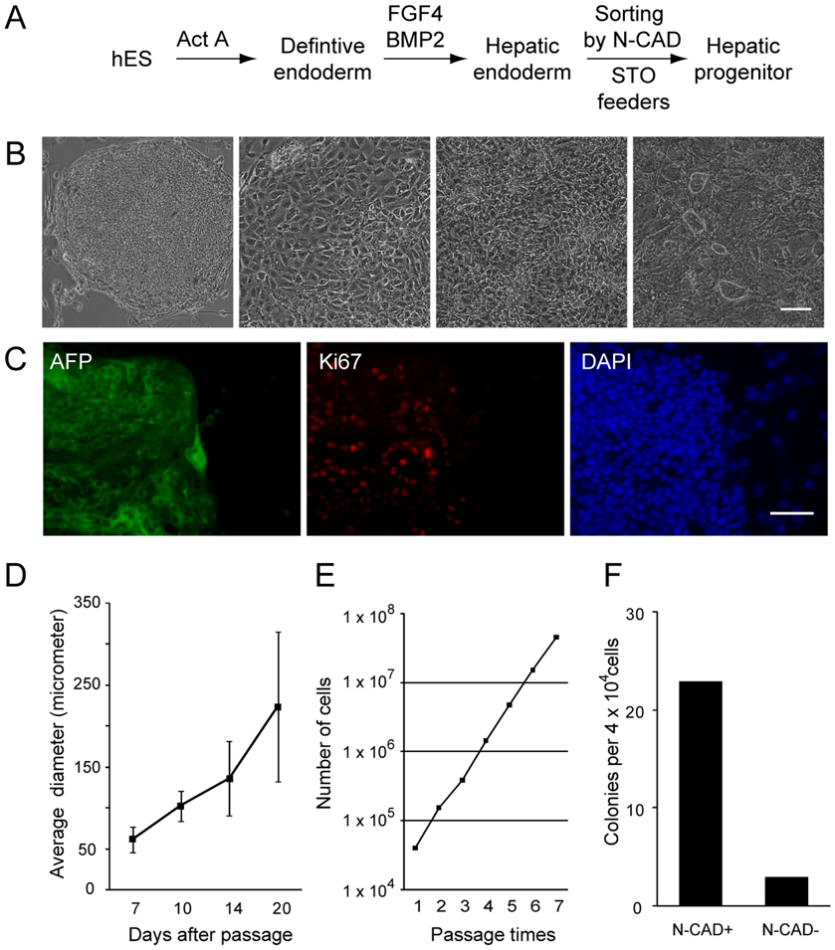
Scheme, cell morphology change, and the proliferation capacity of hepatic progenitor cells derived from hES cells. (A) Hepatic endoderm cells generated by two-step differentiation from hES cells were purified using N-cadherin and further cultured on STO feeders to induce hepatic progenitor cell colony formation. (B) Cell morphology changes during the procedure. (C) Hepatic progenitor cells were highly proliferative and expressed the proliferation marker Ki67. Scale bar = 50 µm. (D) Growth kinetics of the hepatic progenitor colonies. Data represent the mean±s.d. for 8–10 clones selected at random from passage 7. (E) Cumulative growth curve for the hepatic progenitor cells. The number of initial feeder cells was deducted to obtain the final count. (F) Hepatic progenitor colony-forming potential of the N-cadherin^+^ and N-cadherin^−^ populations. The experiments were performed three times, and a representative result is shown. N-CAD = N-cadherin.

Many culture conditions for generating hepatic progenitor cells were tested by adding different growth factors, culturing on different extracellular matrices, and co-culturing with several different feeder cells, including mouse fibroblast cells, STO cells, NIH-3T3 cells, human umbilical vein endothelia cells, and ECV endothelial cells. We found that when hES cell**–**derived hepatic endoderm cells were plated on mitomycin-treated murine embryonic stromal feeders (STO) and cultured in hepatic progenitor expansion medium —a serum-free medium optimized for the proliferation of progenitor cells from rat hepatocytes [18]—parenchymal cell colony appeared (Figure 3A and B). As control, no colonie could be generated when mitomycin-treated feeders were cultured alone under the same conditions. The colonies had compact, sharp boundary morphologies. In contrast to hepatic endoderm cells, which were nonviable or lost hepatic character after passage, the colonies continued to expand. Immunofluorescence staining using antibodies against human nuclear antigens showed that these colonies were composed of human cells, indicating that they were derived from the hES cells and not the STO cells (Figure S3). We interpret these results to indicate that the colonies corresponded to hES cell**–**derived hepatic progenitor cells. The majority of cells in these colonies expressed the proliferation marker Ki67 (Figure 3C). Moreover, we monitored colony growth by measuring the change in diameter over time, as an increment in colony size could be used as an indirect indication of cell proliferation. By 7 days after passaging on STO feeders, the hepatic progenitor cells formed typical colonies that were 62.0±15.4 µm in diameter; by 20 days, these colonies had reached 225.4±92.0 µm, indicating slow and stable cell growth (Figure 3D). The cultures were routinely split at a ratio of 1∶2 or 1∶3 (see Materials and Methods) for more than twelve passages with a population doubling time of ∼5.4 day, and could be frozen and thawed repeatedly (Figure 3E and data not shown).

**Figure 4:**
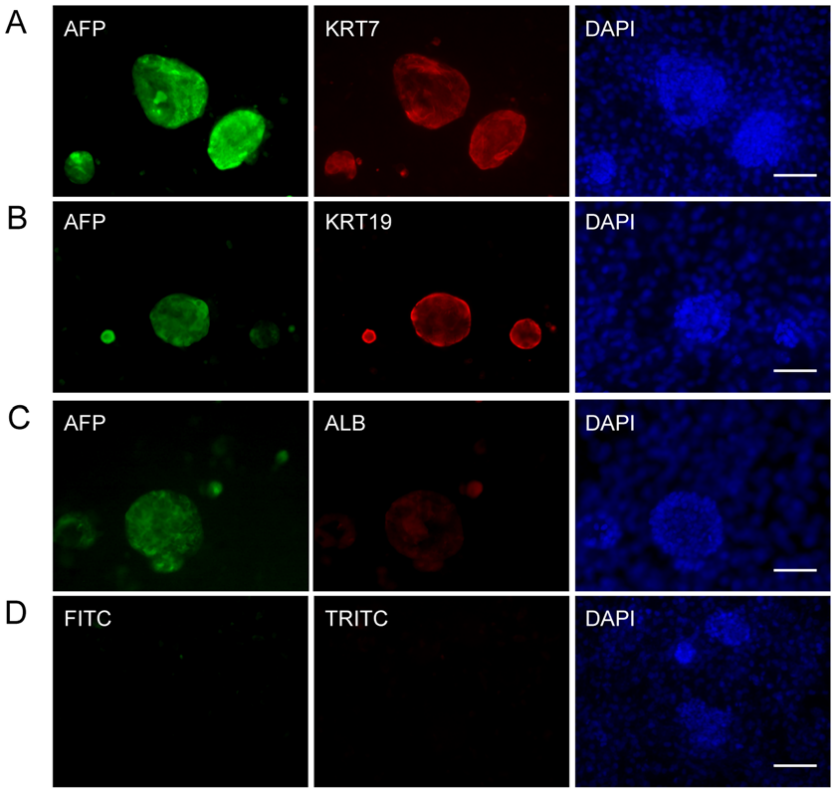
Unique features of hepatic progenitor cells. Hepatic progenitor cells co-expressed AFP and KRT7 (*A*), KRT19 (*B*), and stained faintly for ALB (*C*). (D) Negative control. Scale bar = 50 µm.

To characterize the hES cell**–**derived hepatic progenitor cells, we assessed the marker gene expression using immunofluorescence. These hES cell**–**derived hepatic progenitor cells expressed the early hepatic lineage marker AFP, but demonstrated faint or no expression of the mature hepatocyte marker ALB (Figure 4 A and C). The colonies also expressed the bile duct lineage marker KRT19 and KRT7 (Figure 4 A and B). Moreover, they were positive for the putative hepatic progenitor cell markers EpCAM and CD133 (Figure S4).

To compare the difference in hepatic progenitor generation potential between cells from the N-cadherin^+^ hepatic endoderm population and the N-cadherin^−^ cell population after hepatic fate determination, we cultured the N-cadherin^−^ cell population under the same conditions and found that the number of colonies yielded was at least 6-fold lower than that obtained from the N-cadherin^+^ population (Figure 3F). In addition, these colonies lost rapidly during passage, suggesting that they had little proliferative capacity. This result further supported the use of N-cadherin as a marker to purify hepatic endoderm for the generation of hepatic progenitor cells.

### hES cell–derived hepatic progenitor cells exhibit the potential for differentiation into hepatocyte-like cells

**Figure 5:**
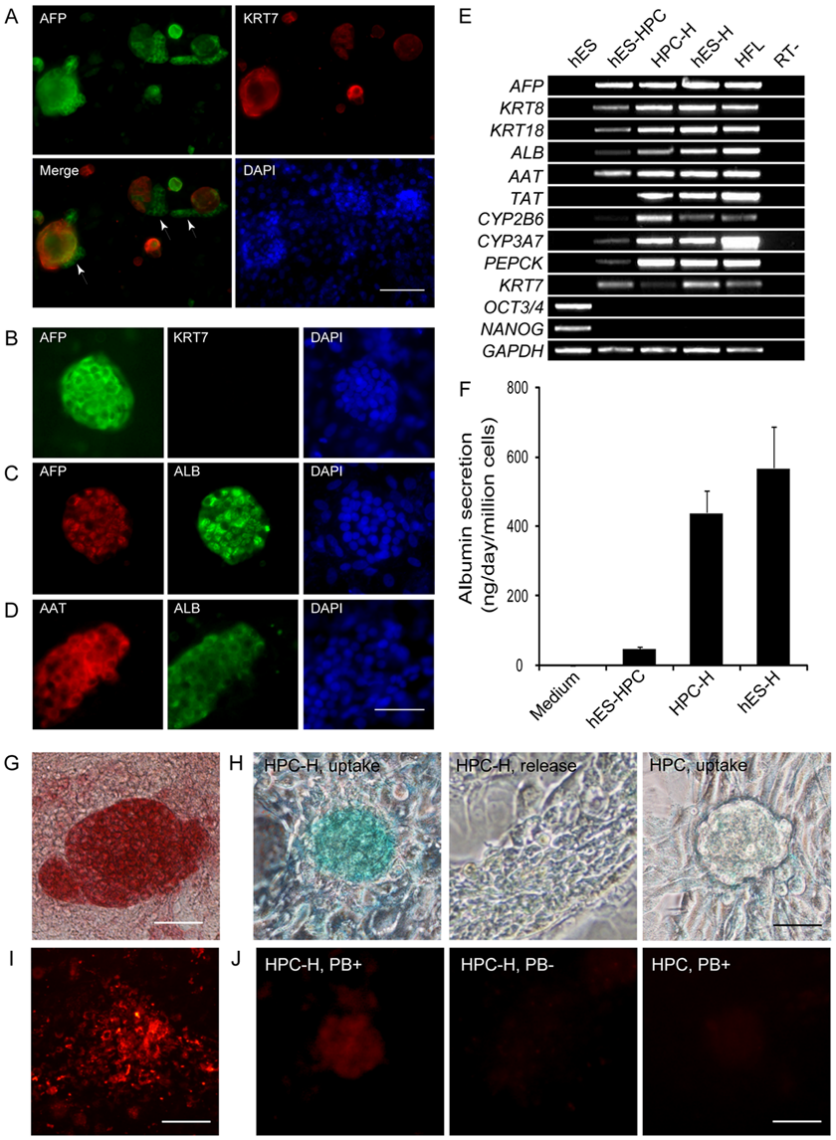
Differentiation of hepatic progenitor cells into hepatocyte-like cells. (A) AFP^+^KRT7^−^ cells were spontaneously generated during culturing of hepatic progenitor cells. Scale bar = 100 µm. (B–D) Hepatic progenitor cells could be induced into KRT7-negative (*B*), AFP (*B* and *C*), ALB (*C*), and AAT-positive (*D*) hepatocyte-like cells. Scale bar = 50 µm. (E) RT-PCR analyses of the mRNA expression of marker genes revealed the similarity of the hepatocyte-like cells differentiated from hepatic progenitor cells to those directly differentiated from hES cells. hES-HPC, hES cell–derived hepatic progenitor cells; HPC-H, hepatocyte-like cells differentiated from hES cell–derived hepatic progenitor cells; hES-H, hepatocyte-like cells directly differentiated from hES cells; HFL, human fetal liver cells. (F) Albumin secretion was determined in vitro by ELISA (n = 7). (G) The PAS assay indicated the cytoplasmic glycogen storage (dark red) ability of these hepatocyte-like cells. (H) ICG taken analysis of the differentiated hepatocyte-like cells (*left*) and undifferentiated hES cell-derived hepatic progenitor cells (*right*). Six hours later, ICG was excluded from the differentiated hepatocyte-like cells which had taken ICG (*middle*). (I) Fluorescence microphotographs showed the uptake of Dil-Ac-LDL by hepatocyte-like cells. (J) PROD assay of the differentiated cells with (*left*) or without (*middle*) PB induction and the undifferentiated hepatic progenitor cells with PB induction (*right*). PB, phenobarbital sodium. Scale bar = 50 µm.

When hES cell**–**derived hepatic progenitor cells were maintained in hepatic progenitor expansion medium, some cells at the periphery of the colonies erupted. In contrast with the AFP^+^ KRT7^+^ progenitor cells, these erupted cells differentiated into AFP^+^ KRT7^−^ cells, indicating spontaneous hepatocyte differentiation potential (Figure 5A). To further confirm this hepatocyte differentiation potential of hES cell**–**derived hepatic progenitor cells, we used HGF and OSM to promote maturation into hepatocytes as reported previously [7]. After 5 days of HGF treatment followed by another 5 days of OSM treatment in hepatocyte culture medium (HCM), we evaluated the expression of hepatocyte markers in the induced cultures by immunofluorescence staining. The induced clusters lost their expression of the cholangiocyte marker KRT7 while maintained the expression of AFP and began to express ALB (Figure 5B and C), which was only faintly expressed in hepatic progenitor cells. Furthermore, most of the ALB-expressing cells exhibited positive AAT staining (Figure 5D). Approximately 20–30% cells differentiated from the hES cell**–**derived hepatic progenitor cells expressed ALB and AAT, which was less efficient than those differentiated directly from hES cells (approximately 50%) [7].

RT-PCR analyses revealed that the expression of genes characteristic of hepatocytes, including *ALB*, *PEPCK*, *AAT*, *TAT*, and two members of the cytochrome P450 superfamily, *CYP3A7* and *CYP2A6*, were increased in the differentiated hepatocyte-like cells comparing with the hepatic progenitor cells; while the expression of ductal marker *KRT7* was decreased (Figure 5E). The gene expression profiles of the hepatocyte-like cells differentiated from hepatic progenitor cells, including *AFP, KRT8, KRT18* and the functional markers mentioned above, were similar with those obtained by our published direct differentiation protocol from hES cells (Figure 5E). Moreover, undifferentiated hES cells markers *OCT3/4* and *NANOG* were expressed neither in the expandable hepatic progenitor cells nor in the hepatocyte-like cells (Figure 5E), which suggested N-CAD derived population did not contaminate undifferentiated hES cells.

To test whether the induced cells possessed hepatocyte functions, we performed a panel of assays on the hepatocyte-like cells differentiated from hES cell**–**derived hepatic progenitor cells. Human albumin secretion of the hepatic progenitor cells was 48.8±5.5 ng/day/million cells by ELISA, but increased dramatically to 439.5±63.5 ng/day/million cells after hepatocyte induction, which was closed to that of the hepatocyte-like cells directly differentiated from hES cells (Figure 5F). We also assayed for glycogen storage of the differentiated cells by Periodic acid Schiff staining on the hepatocyte-like cells. Most of the differentiated cells within the clusters stained red, demonstrating a glycogen storage function (Figure 5G). The uptake and release of indocyanine green (ICG) were used to check the hepatocytes differentiated form hepatic progenitor cells. The differentiated hepatocyte-like cells could uptake ICG from medium and exclude the absorbed ICG six hours later. In contrast, uninduced hepatic progenitor cells did not take up any ICG (Figure 5H). We also confirmed that the differentiated hepatocyte-like cells were capable of taking up Dil-labeled acetylated low-density lipoprotein (Dil-Ac-LDL) (Figure 5I). Furthermore, to analyze the detoxify ability of the differentiated hepatocyte-like cells, we evaluated the cytochrome p450 activity by a PROD assay. When the hepatocyte-like cells cultured in the absence of phenobarbital sodium induction, only a few cells exhibited weak PROD activity (Figure 5J); while after incubation with inducer phenobarbital sodium, the PROD activity was increased, indicating that the induced hepatocyte-like cells possessed inducible P450 activity. In the control experiment, few hepatic progenitor cells had PROD activity even in the presence of phenobarbital sodium induction (Figure 5J). Taken together, all these results indicated that the hES cell**–**derived hepatic cells had the potential for differentiation into hepatocyte-like cells.

### hES cell–derived hepatic progenitor cells exhibit the potential for differentiation into cholangiocyte-like cells

**Figure 6:**
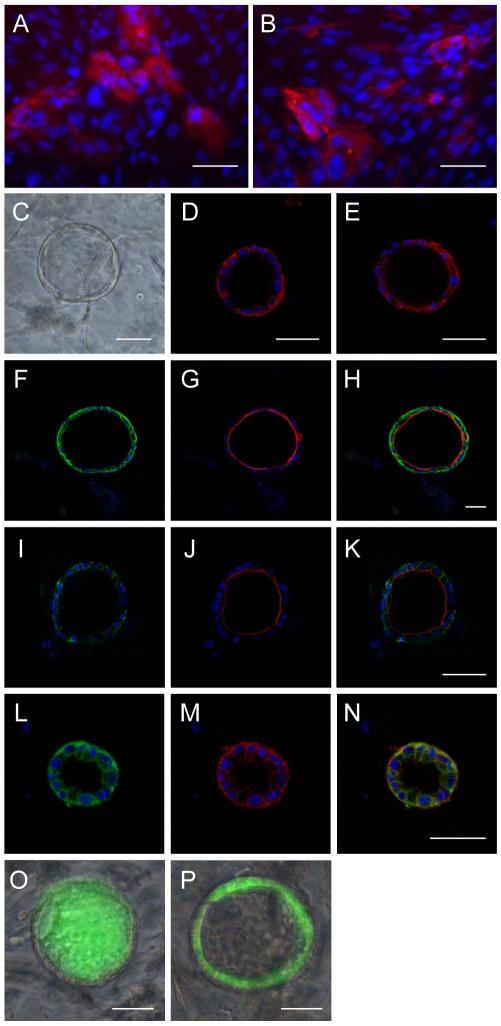
Differentiation of hepatic progenitor cells into cholangiocyte-like cells. (A–B) Hepatic progenitor cells could be induced into KRT7 (*A*) and KRT19-positive (*B*) cholangiocyte-like cells. (C) Morphology of the hepatic progenitor cell–derived ductal cysts formed in a 3D culture system. (D–E) Co-immunofluorescence staining for AFP and cholangiocyte markers. The cysts were positive for KRT19 (*D*, red) and KRT7 (*E*, red), but were negative for AFP (*E*, green). (F–N) Localization of epithelial polarity and ductal markers. β-catenin (*F*), E-cadherin (*I*), and Intergrin α_6_ (*L*) localized to the basolateral membrane, while F-actin (*G* and *J*) localized to the apical membrane. The ductal marker KRT19 localized to both the basolateral and apical membranes (*M*). Merged images are shown in *H*, *K*, and *N*. (O) Transport of rhodamine 123 into the central lumen of a cyst. (P) The MDR inhibitor verapamil blocks rhodamine 123 transport. Scale bar = 50 µm.

To test the ductal differentiation potential of hES cell**–**derived hepatic progenitor cells, we cultured them in William's E medium for 7 days on plates coated with matrigel, which has been reported to promote hepatic progenitor cells differentiation into cholangiocytes [19]. Immunofluorescence indicated that KRT19- and KRT7-positive, AFP-negative cells appeared (Figure 6A and B), suggesting that the hES cell**–**derived hepatic progenitor cells had differentiated into cholangiocyte-like cells. Furthermore, we differentiated hES cell**–**derived hepatic progenitor cells in a three-dimensional (3D) culture system in which cells were grown in a gel consisting of matrigel and collagen I. This system has been widely used to investigate the mechanisms underlying polarization and tubulogenesis of epithelial cells [20], and it can also be used to investigate the epithelial polarity of differentiated cholangiocytes [21]. When cultured in the 3D system for 7 days, differentiated hepatic progenitor cells formed round cysts with a central luminal space surrounded by a monolayer of cells (Figure 6C). We examined the expression of lineage markers for liver epithelial cells. KRT7 and KRT19, two conventional markers of cholangiocytes, were detected in the surrounding monolayer cells, whereas the hepatic lineage marker AFP was undetectable (Figure 6D and E). We then checked whether these cells acquired apicobasal polarity as cholangiocytes and found that β-catenin was localized to the basolateral cell surfaces and F-actin bundles were enriched in the inner layer of the lumen, indicating the presence of apicobasal polarity (Figure 6F-H). E-cadherin and intergrin α_6_ were also localized to the basolateral region (Figure 6I-N). To determine whether the differentiated cholangiocyte-like cells possessed a secretory function, we analyzed the function of MDR, which is an ATP-dependent transmembrane export pump that may mediate the biliary secretion of cationic organic solutes [22]. When the cysts were incubated in the presence of rhodamine 123, the fluorescence intensity was much greater inside the luminal space than in the surrounding cells (Figure 6O). In addition, rhodamine 123 was trapped inside cells and was not transported into the central lumen in the presence of 10 M verapamil, an MDR inhibitor (Figure 6P), indicating that the transport of rhodamine 123 depended on functional MDR in the apical domain. Taken together, these data demonstrated that the differentiated cells derived from hepatic progenitor cells show great similarity to cholangiocytes *in vivo*.

## Discussion

In this study, we demonstrated for the first time that hES cells could be differentiated into hepatic progenitor cells. These hES cell**–**derived hepatic progenitor cells could maintain their proliferation capacity for more than 100 days of culture *in vitro*, while maintaining their differentiation potential into both hepatocyte-like and cholangiocyte-like cells. After they were expanded, hepatic progenitor cell cultures could undergo differentiation into hepatocyte-like cells that expressed ALB and AAT and stored glycogen, or into cholangiocyte-like cells that formed duct-like cyst structures, expressed KRT7 and KRT19 and acquired epithelial polarity.

We found that N-cadherin can be used as a surface marker for the enrichment of hepatic endoderm cells differentiated from hES cells. Previous studies tracking hepatic endoderm cells from mixed hES cell derivates were limited to using an ES cell line with a reporter gene targeted to AFP, Foxa2 or another hepatic-specific locus[2], [3], [5], [23]. To our knowledge, the purification of hepatic endoderm cells from derivates of an unmanipulated ES cell line had not been reported. In the present study, we found that N-cadherin expression specifically matched AFP expression after the generation of hepatic endoderm cells (Figure 1B). These results are consistent with previous reports describing the N-cadherin expression pattern during mouse and human liver development [24]–[27]. In mouse embryos, N-cadherin is first detected by immunohistochemistry in the liver at E10.5 and is observed throughout the liver from E12.5 to adulthood. In the human liver, N-cadherin is expressed during the fetal stage and continues to be expressed in adult hepatocytes. In addition, N-cadherin is uniquely expressed in hepatic endoderm cells, but not in undifferentiated hES cells in this differentiation system [28]. Therefore, compared with other reported surface markers of fetal liver such as EpCAM and CD49f which are also expressed in undifferentiated ES cells [29], N-cadherin can be used as a specific surface marker for hepatic-committed cells to exclude undifferentiated ES cells under conditions that promote hepatic lineage differentiation.

To date, the majority of studies have focused on the differentiation of hES cells toward mature hepatocytes, and there have been no previous reports investigating early hepatic cell proliferation potential during the ES cell differentiation process. In this study, we managed to couple the ability to generate hES cell**–**derived hepatic progenitor cells with the capacity to expand this population *in vitro*. We generated hES cell**–**derived hepatic progenitor cells that could be passaged more than 12 times at a 1∶2 or 1∶3 split ratio and could be cryopreserved and thawed repeatedly. The high purity, availability, and bipotential of hES cell**–**derived hepatic progenitor cells will provide the basis for future therapeutic efforts in preclinical animal models of disease.

The hES cell**–**derived hepatic progenitor cells obtained in the present study appear to represent a population of cells similar to those directly isolated from human fetal liver. Like the hepatic stem cells and hepatoblast cells recently identified in fetal and adult human livers by Reid's group [12], [30], our hES cell**–**derived hepatic progenitor cells could also be maintained on STO-feeder cells in serum-free medium. Moreover, both of these cells expressed AFP and CK19, as well as the surface makers EpCAM and CD133, suggesting similar developmental origin.

Previous studies have reported a few biliary lineage cells in close proximity to hepatocytes during the differentiation process [7]; however, the differentiation of cholangiocytes from hES cells has been detected only on the basis of the expression of KRT7 and KRT19. It is difficult to determine cholangiocyte differentiation simply by analyzing gene expression, because only a few markers are available and they are not closely related to cholangiocyte function. In this study, we report a method to direct the differentiation of hES cells into cholangiocyte-like cells through the progenitor cell stage. Moreover, we employed multiple standards including function assays to identify the biliary identity of these differentiated cells. After induction, the differentiated biliary cells (1) esxpressed KRT7 and KRT19, two conventional markers of cholangiocytes, (2) formed a lumen with epithelial polarity, demonstrating *in vitro* tubulogenesis, and (3) transported a fluorescent dye, indicating functional MDR expression (Figure 6). In conclusion, these multiple tests together showed that the differentiated cells were similar to functional cholangiocytes.

In summary, this is the first report of the direct generation of proliferative and bipotential hepatic progenitor cells differentiated from hES cells. Because the generation process of hepatic progenitor cells during *in vitro* differentiation mimics the development of progenitor cells *in vivo*, these hES cell**–**derived hepatic progenitor cells could be used as an *in vitro* model for studying the early events of hepatic progenitor cell development. In addition, they displayed proliferation and bipotentiality, which will facilitate studies of the molecular mechanisms of hepatic stem/progenitor cell origin, self-renewal and differentiation *in vitro*.

## Materials and Methods

### Culture and differentiation of hES cells into hepatic endoderm cells

The human ES cell line H1 was obtained from WiCell Research Institute (Madison, WI) and maintained as described previously [7]. For hepatic differentiation, hES cells were induced following the first two steps of a previously established stepwise protocol [7].

### Flow cytometry and cell sorting

Day 8 hepatic endoderm cells were dissociated by treatment with 0.25% trypsin containing 2 mM Ca^2+^ instead of EDTA. The resulting cells were stained with anti-N-cadherin (Clone GC4, Sigma-Aldrich, St Louis, MO) or mouse IgG (Sigma-Aldrich), followed by phycoerythrin-conjugated anti-mouse IgG antibodies (Jackson ImmunoResearch, West Grove, PA) in phosphate-buffered saline containing 1 mg/ml albumin. Cells were sorted with a MoFlo cell sorter (Dako Cytomation, Carpinteria, CA) and the data were analyzed using Summit Software, version 4.0 (Dako Cytomation).

### Culture, expansion and differentiation of hepatic progenitor cells

Hepatic endoderm cells were plated on a monolayer of mitomycin-treated STO feeder cells in hepatic progenitor expansion medium [18]. Colonies were observed within 7–10 days. The hepatic progenitor cells were passaged at a ratio of 1∶2 or 1∶3 every 8–12 days. For hepatocyte differentiation, hepatic progenitor cells were cultured in HCM containing 20 ng/ml HGF (Peprotech, Rocky Hill, NJ) for 5 days, and 10 ng/ml OSM (R&D System, Minneapolis, MN) plus 0.1 µM dexamethasone (Sigma-Aldrich) for the next 5 days. For cholangiocyte differentiation, hepatic progenitor cells were plated in Matrigel-coated (Becton-Dickinson, Bedford, MA, 1∶30) cell culture plates for attachment, and then incubated with William's E medium (Sigma-Aldrich) supplemented described previously [31]. For biliary differentiation in a 3D system, 1×10^5^ hepatic progenitor cells were suspended in a mixture of 240 µl of type-I collagen gel (R&D System), 400 µl of Matrigel and 360 µl of William's E medium with supplements. After incubation at 37°C for 2 hours to solidify the gel, 800 µl of William's E medium with supplements was added on the top of the solid gel and changed every 2 days.

### Immunofluorescence

Cells or tissue sections were fixed and stained as described previously [7]. Optimized concentrations of primary antibodies are shown in Table S2. FITC or TRITC-conjugated secondary antibodies were purchased from Jackson ImmunoResearch. F-actin was detected with AlexaFluor 546-conjugated phalloidin (Invitrogen, Carlsbad, CA) at a dilution of 1∶200.

### RT-PCR and qPCR analysis of gene expression

Total RNA was isolated from cells using TRIzol Reagent (Invitrogen) and genomic DNA was removed using TURBO DNA-free Kit (Ambion, Austin, TX) according to the manufacturer's protocol. RT-PCR and qPCR were performed as previously described [7]. Primer sequences and annealing temperatures are shown in Table S3 and S4.

### Assays of hepatocyte function

PAS stain for glycogen, uptake of LDL, ICG uptake and PROD assay were performed as described previously [7]. For the measurement of albumin secretion, the human albumin content in the supernatant was determined by Human Albumin ELISA Quantitation kit (Bethyl Laboratory, Montgomery, TX) under the manufacturer's instructions. The albumin secretion was normalized to the cell count.

### Assay for transport of fluorescent dye

Hepatic progenitor cells were cultured in a Chambered Coverglass (Nalgene Nunc, Naperville, IL) for biliary differentiation in a 3D system for 7 days. Further assays to assess the transport of rhodamine 123 and R-(+)-verapamil effect were performed as described proviously [21].

## Supporting Information

Figure S1 Flow cytometry analysis of hepatic endoderm cells. Day 8 cells were dissociated and stained with anti-N-cadherin and anti-AFP antibody.
(2.30 MB TIF)

Figure S2 Immunofluorescence staining of post-sorted N-cadherin^+^ and N-cadherin^−^ cells. *Left*, AFP-expressing (green) cells were enriched in N-cadherin^+^ cells by cell sorting. *Right*, AFP-expression is hardly detected in N-cadherin^−^ cell population. Cell nuclei are stained with DAPI (blue). Scale bar = 50 µm.
(3.50 MB TIF)

Figure S3 Immunofluorescence staining demonstrated the human cell origin of colonies yielded on STO feeder cells. *Upper panel*, colonies were stained using antibodies against AFP and human nucleus (HuNu). *Lower panel*, STO feeder cells stained as control. Cell nuclei are stained with DAPI (blue). Scale bar = 100 µm.
(3.54 MB TIF)

Figure S4 Flow cytometry analysis of putative hepatic progenitor marker expression in hES cell−derived hepatic progenitor cells. A substantial portion of hepatic progenitor cells cultured on the feeder cells showed the expression of EpCAM and CD133. As control, STO feeder cells did not express either EpCAM or CD133.
(4.44 MB TIF)

Table S1 Relationship between expression of AFP and some surface proteins in day 8 differentiation.
(0.04 MB DOC)

Table S2 Primary antibodies and dilution factors.
(0.04 MB DOC)

Table S3 Semiquantitative RT-PCR primers.
(0.04 MB DOC)

Table S4 Quantitative RT-PCR primers.
(0.04 MB DOC)
