# Supplementary figures and images for: Derivation and Characterization of Hepatic Progenitor Cells from Human Embryonic Stem Cells
Source: PLoS One. 2009 Jul 31;4(7):e6468. doi: 10.1371/journal.pone.0006468 (PMC2714184; doi:10.1371/journal.pone.0006468)

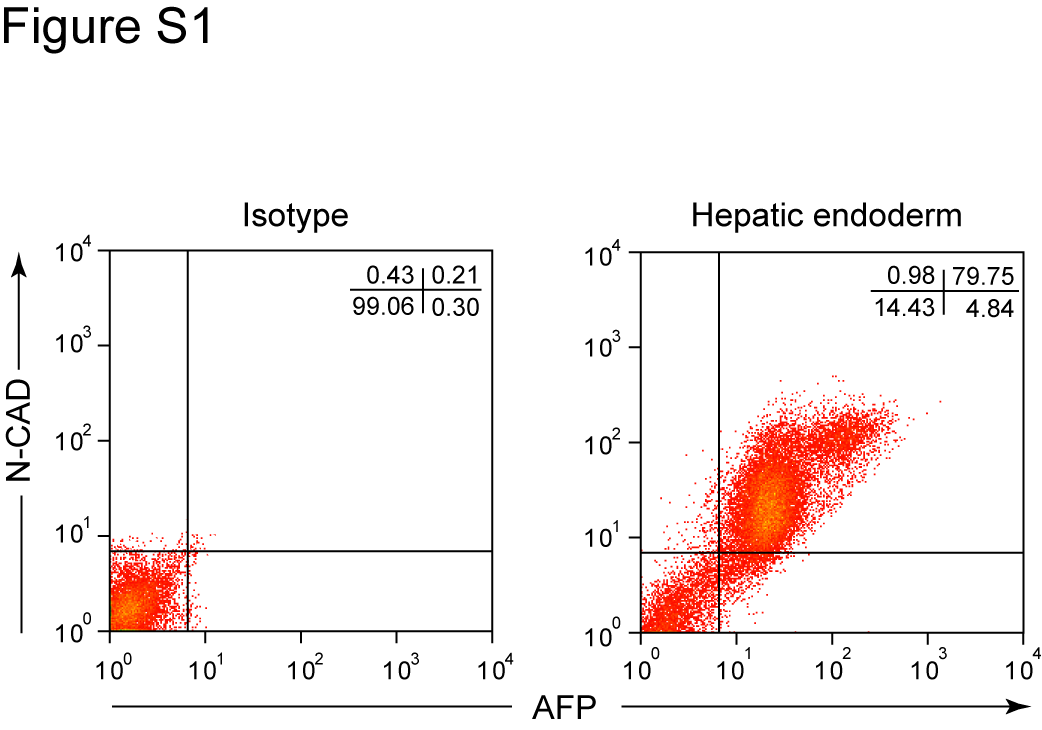

Supplement: Figure S1 — Flow cytometry analysis of hepatic endoderm cells. Day 8 cells were dissociated and stained with anti-N-cadherin and anti-AFP antibody. (2.30 MB TIF) [file pone.0006468.s001.tif]

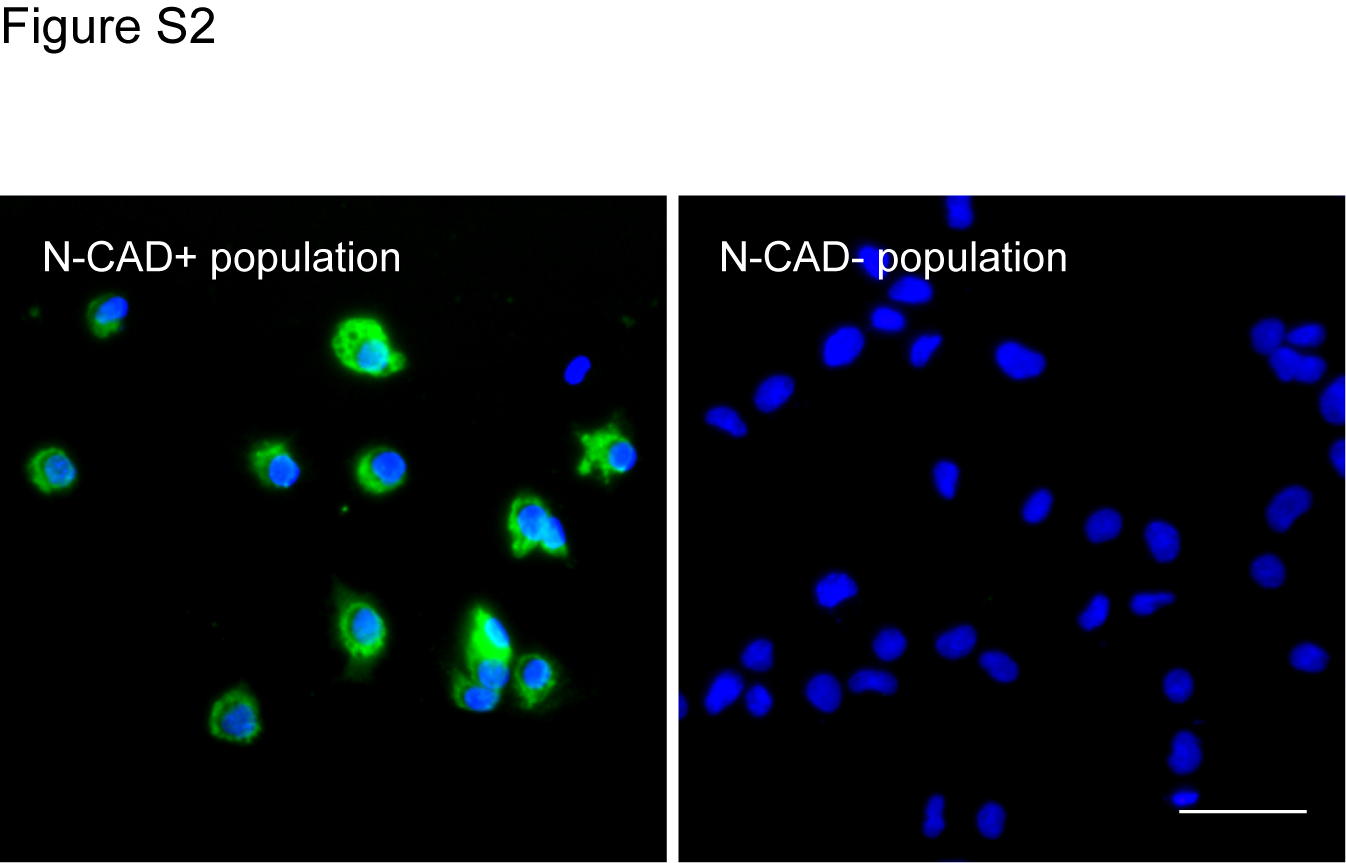

Supplement: Figure S2 — Immunofluorescence staining of post-sorted N-cadherin+ and N-cadherin− cells. Left, AFP-expressing (green) cells were enriched in N-cadherin+ cells by cell sorting. Right, AFP-expression is hardly detected in N-cadherin− cell population. Cell nuclei are stained with DAPI (blue). Scale bar = 50 µm. (3.50 MB TIF) [file pone.0006468.s002.tif]

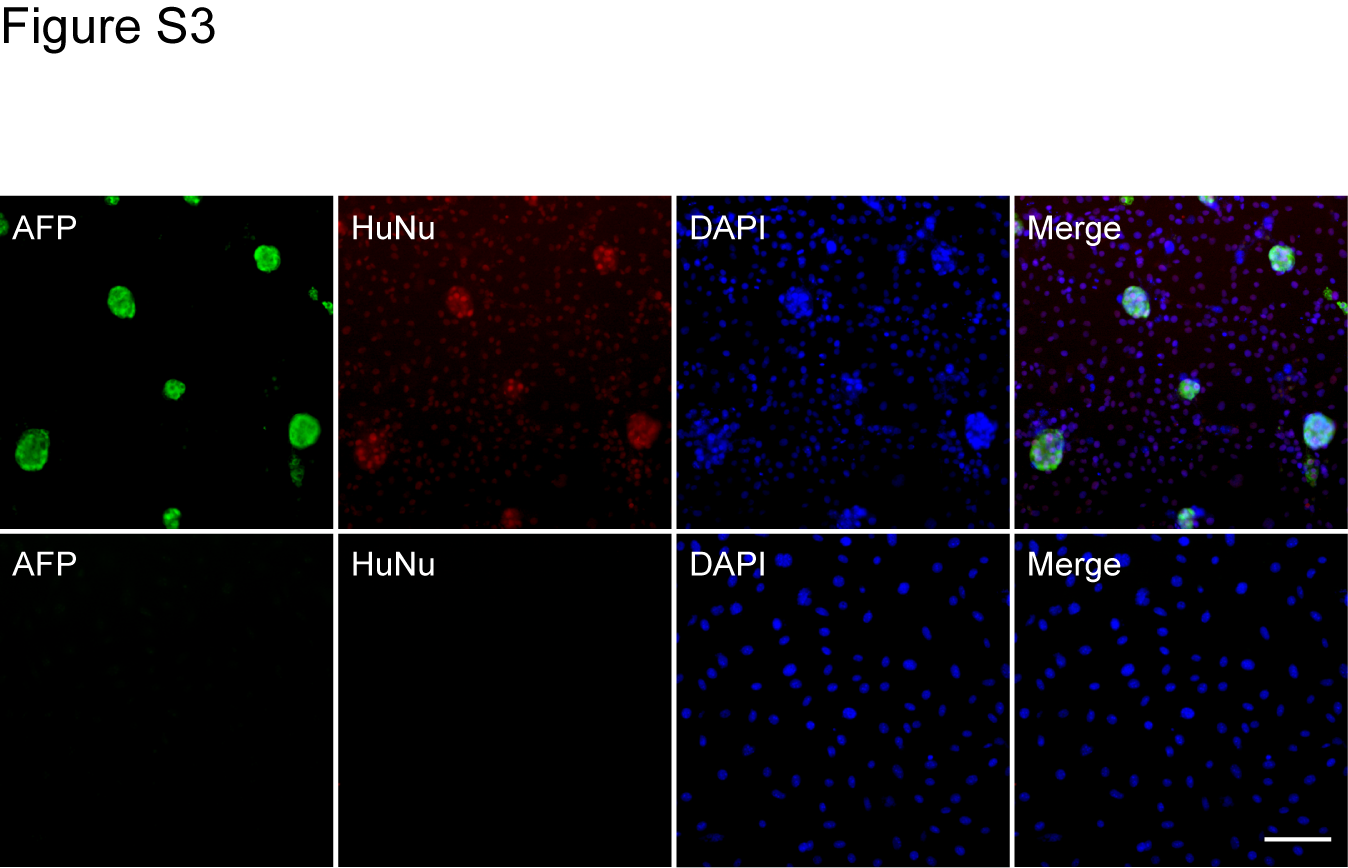

Supplement: Figure S3 — Immunofluorescence staining demonstrated the human cell origin of colonies yielded on STO feeder cells. Upper panel, colonies were stained using antibodies against AFP and human nucleus (HuNu). Lower panel, STO feeder cells stained as control. Cell nuclei are stained with DAPI (blue). Scale bar = 100 µm. (3.54 MB TIF) [file pone.0006468.s003.tif]

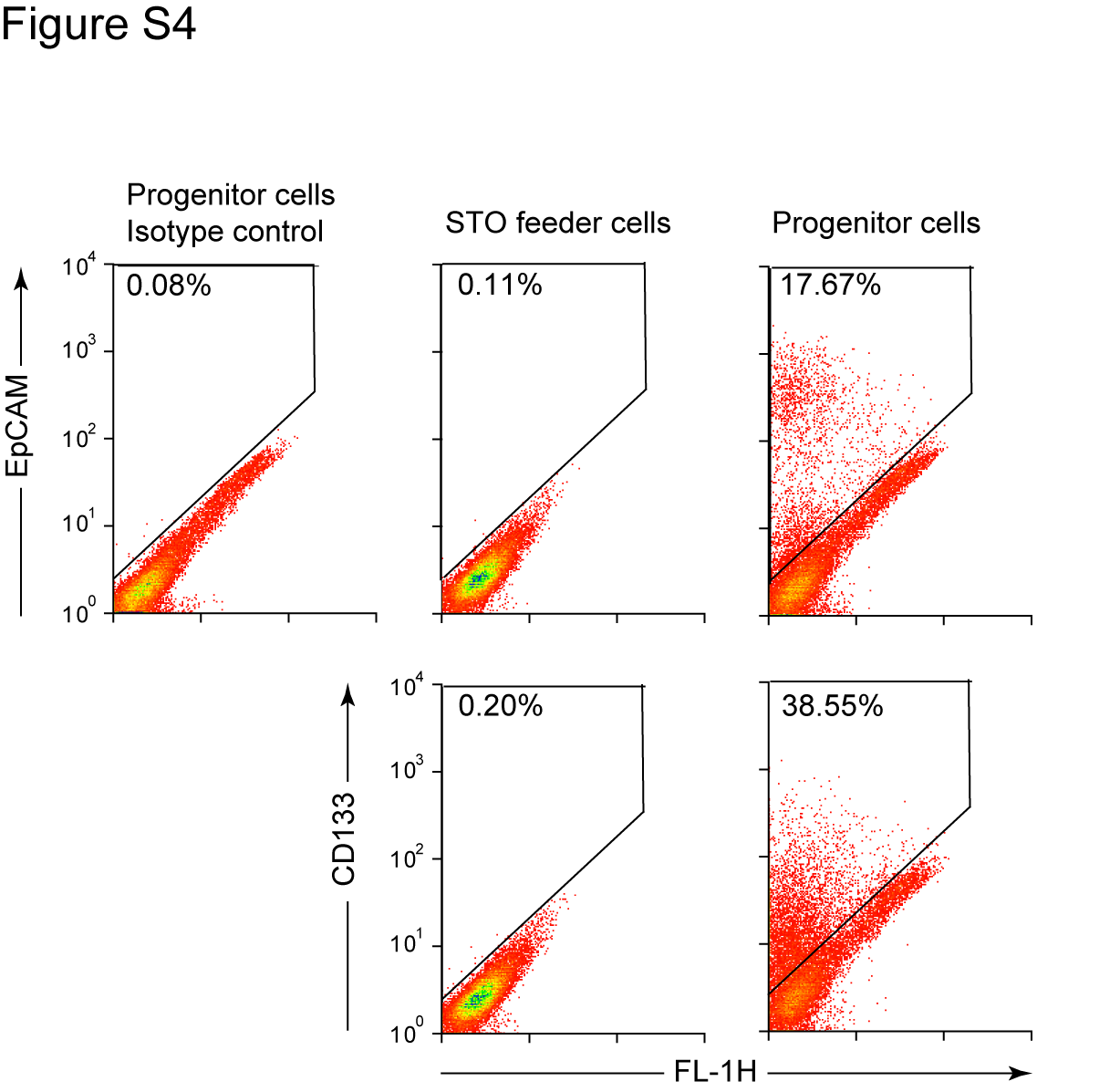

Supplement: Figure S4 — Flow cytometry analysis of putative hepatic progenitor marker expression in hES cell−derived hepatic progenitor cells. A substantial portion of hepatic progenitor cells cultured on the feeder cells showed the expression of EpCAM and CD133. As control, STO feeder cells did not express either EpCAM or CD133. (4.44 MB TIF) [file pone.0006468.s004.tif]
