# Supplementary material for: Derivation and Characterization of Hepatic Progenitor Cells from Human Embryonic Stem Cells
Source: PLoS One. 2009 Jul 31;4(7):e6468. doi: 10.1371/journal.pone.0006468 (PMC2714184; doi:10.1371/journal.pone.0006468)
Supplement: Table S1 — Relationship between expression of AFP and some surface proteins in day 8 differentiation. (0.04 MB DOC) [file pone.0006468.s005.doc]

**Table S1. Relationship between expression of AFP and some surface proteins in day 8 differentiation.**

| **Surface proteins** | **Detection methods** | **Relationship with AFP expression** |
| --- | --- | --- |
| CD29 | Flow cytometry | Partially overlap |
| CD34 | Flow cytometry | No overlap |
| CD49f | Flow cytometry | Partially overlap |
| CD133 | Flow cytometry | Partially overlap |
| Thy-1 | Flow cytometry | No overlap |
| c-kit | Flow cytometry | No overlap |
| c-met | Immunostaining | No overlap |
| N-cadherin | Immunostaining | Specifically match |
| E-cadherin | Immunostaining | No overlap |
| N-CAM | Immunostaining | No overlap |
| EpCAM | Flow cytometry | Partially overlap |

All antibodies were purchased from Becton-Dickinson, except c-Met which was purchased from Millipore, Billerica, MA.
