# Supplementary material for: Derivation and Characterization of Hepatic Progenitor Cells from Human Embryonic Stem Cells
Source: PLoS One. 2009 Jul 31;4(7):e6468. doi: 10.1371/journal.pone.0006468 (PMC2714184; doi:10.1371/journal.pone.0006468)
Supplement: Table S2 — Primary antibodies and dilution factors. (0.04 MB DOC) [file pone.0006468.s006.doc]

**Table S2. Primary antibodies and dilution factors.**

| **Antibody** | **Origin** | **Company** | **Work Dilution** |
| --- | --- | --- | --- |
| N-cadherin | Mouse | Becton-Dickinson | 1:200 |
| AFP | Rabbit | Dako Cytomation | 1:200 |
| AFP | Mouse | Invitrogen | 1:200 |
| ALB | Rabbit | Dako Cytomation | 1:500 |
| ALB | Mouse | Sigma-Aldrich | 1:400 |
| CK7 | Mouse | Invitrogen | 1:200 |
| CK19 | Mouse | Invitrogen | 1:200 |
| E-Cadherin | Mouse | R&D systems | 1:100 |
| β-catenin | Mouse | Becton-Dickinson | 1:200 |
| Intergrin α_6_ | Rat | Becton-Dickinson | 1:200 |
| Ki67 | Mouse | Invitrogen | 1:200 |
| BrdU | Mouse | Invitrogen | 1:150 |
| AAT | Rabbit | Invitrogen | 1:200 |
