# Supplementary material for: Derivation and Characterization of Hepatic Progenitor Cells from Human Embryonic Stem Cells
Source: PLoS One. 2009 Jul 31;4(7):e6468. doi: 10.1371/journal.pone.0006468 (PMC2714184; doi:10.1371/journal.pone.0006468)
Supplement: Table S3 — Semiquantitative RT-PCR primers. (0.04 MB DOC) [file pone.0006468.s007.doc]

**Table S3. Semiquantitative RT-PCR primers.**

| **Gene** | **Primer sequence (forward and reverse)** | **TM (°C)** | **Product Length (bp)** |
| --- | --- | --- | --- |
| *GAPDH* | AATCCCATCACCATCTTCC  CATCACGCCACAGTTTCC | 56 | 382 |
| *PEPCK* | CTTCGGCAGCGGCTATGGT  TGGCGTTGGGATTGGTGG | 50 | 383 |
| *AFP* | TTTTGGGACCCGAACTTTCC  CTCCTGGTATCCTTTAGCAACTCT | 56 | 451 |
| *ALB* | GGTGTTGATTGCCTTTGCTC  CCCTTCATCCCGAAGTTCAT | 56 | 502 |
| *AAT* | ggacctctgtctcgtcttgg  gctctgatttggggttgtgt | 60 | 183 |
| *TAT* | CCCCTGTGGGTCAGTGTT  GTGCGACATAGGATGCTTTT | 56 | 345 |
| *CYP2B6* | AGGGAGATTGAACAGGTGATT  GATTGAAGGCGTCTGGTTT | 56 | 253 |
| *CYP3A7* | CTATGATACTGTGCTACAGT  TCAGGCTCCACTTACGGTCT | 50 | 455 |
| *KRT8* | GGAGGCATCACCGCAGTAC  TCAGCCCTTCCAGGCGAGAC | 56 | 472 |
| *KRT18* | GGTCTGGCAGGAATGGGAGG  GGCAATCTGGGCTTGTAGGC | 56 | 460 |
| *KRT7* | TCCGCGAGGTCACCATTAAC GCTGCTCTTGGCCGACTTCT | 55 | 218 |
| *OCT3/4* | GAACCGAGTGAGAGGCAACC  ATCCCAAAAACCCTGGCACA | 55 | 457 |
| *NANOG* | TGCCTCACACGGAGACTG  GCTATTCTTCGGCCAGTT | 55 | 353 |
