# Supplementary material for: Derivation and Characterization of Hepatic Progenitor Cells from Human Embryonic Stem Cells
Source: PLoS One. 2009 Jul 31;4(7):e6468. doi: 10.1371/journal.pone.0006468 (PMC2714184; doi:10.1371/journal.pone.0006468)
Supplement: Table S4 — Quantitative RT-PCR primers. (0.04 MB DOC) [file pone.0006468.s008.doc]

**Table S4. Quantitative RT-PCR primers.**

| **Gene** | **Primer sequence (forward and reverse)** | **TM (°C)** | **Product Length (bp)** |
| --- | --- | --- | --- |
| *GAPDH* | TGCACCACCAACTGCTTAGC  GGCATGGACTGTGGTCATGAG | 60 | 87 |
| *AFP* | CCCGAACTTTCCAAGCCATA  TACATGGGCCACATCCAGG | 60 | 101 |
| *ALB* | GCACAGAATCCTTGGTGAACAG  ATGGAAGGTGAATGTTTCAGCA | 60 | 101 |
| *HNF4A* | ACTACATCAACGACCGCCAGT  ATCTGCTCGATCATCTGCCAG | 60 | 103 |
| *CEBPA* | ACAAGAACAGCAACGAGTACCG  CATTGTCACTGGTCAGCTCCA | 60 | 129 |
| *N-CAD* | TAACTGGGCCAGGAGCTGAC  TATCTGCTCGCGATCCAGG | 60 | 101 |
| *FOXA2* | CTGAGCGAGATCTACCAGTGGA  CAGTCGTTGAAGGAGAGCGAGT | 60 | 104 |
